# Alzheimer's Proteins, Oxidative Stress, and Mitochondrial Dysfunction Interplay in a Neuronal Model of Alzheimer's Disease

**DOI:** 10.4061/2010/621870

**Published:** 2010-09-02

**Authors:** Antonella Bobba, Vito A. Petragallo, Ersilia Marra, Anna Atlante

**Affiliations:** Istituto di Biomembrane e Bioenergetica, CNR, Via Amendola 165/A, 70126 Bari, Italy

## Abstract

In this paper, we discuss the interplay between beta-amyloid (A*β*) peptide, Tau fragments, oxidative stress, and mitochondria in the neuronal model of cerebellar granule neurons (CGNs) in which the molecular events reminiscent of AD are activated. The identification of the death route and the cause/effect relationships between the events leading to death could be helpful to manage the progression of apoptosis in neurodegeneration and to define antiapoptotic treatments acting on precocious steps of the death process. Mitochondrial dysfunction is among the earliest events linked to AD and might play a causative role in disease onset and progression. Recent studies on CGNs have shown that adenine nucleotide translocator (ANT) impairment, due to interaction with toxic N-ter Tau fragment, contributes in a significant manner to bioenergetic failure and mitochondrial dysfunction. These findings open a window for new therapeutic strategies aimed at preserving and/or improving mitochondrial function.

## 1. Introduction

Alzheimer's disease (AD) is a common neurodegenerative disorder characterized by altered processing of specific proteins, formation of neurofibrillary tangles, imbalance of redox homeostasis, and degeneration of synapses and neurons. Although the mechanism of neurodegeneration in AD is not clearly understood, several studies presently indicate that apoptosis might occur and contribute to AD onset and progression [1–5]. Though it remains to be determined whether true apoptosis is a necessary event in neurodegeneration, a growing number of studies support the activation of apoptosis in general, and caspases specifically, as an early event that contributes to neurodegeneration and promote the pathological hallmarks associate with AD [6]. 

Transgenic animal models have been useful tools to study AD, but currently many of them do not fully replicate the cascade of amyloid deposition, neurofibrillary tangles, and neurodegeneration that characterize the human disease [7]. Thus, as far as the studies about AD are concerned, the lack of an animal model that sufficiently resembles this disease is the reason why research should still proceed along parallel lines: studies carried out in animal models should be integrated and correlated to ad hoc-devised neuronal models in which the identification of single molecular steps is made possible. 

Rat cerebellar granule neurons (CGNs) are a neuronal model widely used to study events linking apoptosis and neurodegeneration [8, 9] due to the ease of CGN culture production, their high degree of cellular homogeneity, and the findings revealing that during the onset of apoptosis several molecular events reminiscent of AD are activated [10]. 

In this paper, the role of key players of the neuronal apoptotic process is discussed with particular attention to the results obtained in CGNs. The production, effect, and interplay of beta-amyloid (A*β*), Tau protein and its fragments are discussed together with the action of these proteins on mitochondria, and this is integrated in the scenario of CGN apoptosis.

## 2. The Experimental Model of CGNs: A Useful Model to Elucidate Neurodegenerative Mechanism(s)

CGNs survive and differentiate in vitro in the presence of depolarizing concentrations of KCl (25 mM) without additional need for neurotrophic factors [11]. The mechanism of action of KCl is still controversial but, generally, it is believed that the increase in intracellular Ca^2+^ concentration [12, 13] and the activation of mitogen-activated protein kinase (MAPK) [14] induced by depolarization are involved. 

If the serum is removed, and the concentration of KCl is kept below depolarizing levels (K5), the majority of CGNs die by an apoptotic process [12]. Under these conditions, that are equivalent to in vivo deafferentation, neuronal death is initiated and follows a general scheme that has been extensively characterized in recent years (for a review see [15]). The production of reactive oxygen species (ROS) and nitric oxide (NO), the increase in proteasome, antioxidant enzyme, and nitric oxide synthase (NOS) activities, and release of cytochrome *c* (cyt *c*) into the cytosol are some of the main events taking place soon after apoptosis induction in CGNs and for which a cause-effect relationship has been defined. In the early phase of apoptosis, ROS, NO, and cGMP production increases as well as the activities of antioxidant enzymes and NOS [16–20], as the cell's attempt to counteract the ongoing oxidative stress [18]. However, due to superoxide production, cyt *c *is released into the cytosol where it carries out a triple function since it acts (i) as an antioxidant compound and an ROS scavenger, (ii) as a respiratory substrate which can generate the mitochondrial transmembrane potential, and (iii) as the activator of the caspase cascade [21–23]. As a consequence of both NO and superoxide anion production, an increase in the levels of nitrated proteins has been found in the late phase (ranging from 3 to 15 hours after apoptosis induction) [19]. With apoptosis progression, the oxidative damage proceeds, antioxidant enzymes are inactivated by caspases and proteasome [18, 24], and, at the mitochondrial level, the adenine nucleotide translocator (ANT) is progressively impaired thus contributing to the transition pore opening in the late phase of the death process [25, 26]. 

Furthermore, it has been demonstrated that during the onset of apoptosis of CGNs, several molecular events reminiscent of AD are induced. An amyloidogenic process is activated with an increased production of A*β* which initiates a sort of autocrine toxic loop [27]. Contextually to the increase in A*β* deposition, Tau protein, which is the main constituent of AD neurofibrillary tangles, is cleaved by the concerted action of calpain and caspases with the production of toxic fragments [28, 29]. The mechanism of action of a Tau toxic fragment has been elucidated, and ANT has been identified as the specific mitochondrial target of such fragment [30].

## 3. Formation of *A* *β* and Tau Protein Fragments in AD

One of the central points in the physiopathology of AD is the altered function and/or structure of two “Alzheimer's proteins,” namely the amyloid precursor protein (APP) and Tau. 

APP is a membrane glycoprotein, which undergoes complex intracellular trafficking. The biological function of APP is still not fully clear. Roles in cell adhesion, neuronal migration, cell proliferation, neurite outgrowth, axonal transport, neuroprotection, and signal transduction have been proposed [31]. The abnormal cleavage of APP leads to the production of A*β* which is the main component of senile plaques in AD and *per se* can induce neuronal cell death.

Tau is a neuron-specific microtubule-associated protein and a critical component of the neuronal cytoskeleton which progressively disaggregates during apoptosis. The proper function of Tau depends upon a precise equilibrium between different isoforms and its state of phosphorylation. In AD, as well as in other human dementias, Tau undergoes a series of posttranslational changes including abnormal phosphorylation, glycosylation, glycation, and truncation (see [32]), which may render Tau more prone to form aggregated structures, the neurofibrillary tangles, which constitute a major hallmark of AD. Following such aggregation, the microtubules disintegrate, collapsing the neuron's transport system, with consequent altered communication between neurons, eventually ending in cell death.

**Figure 1:**
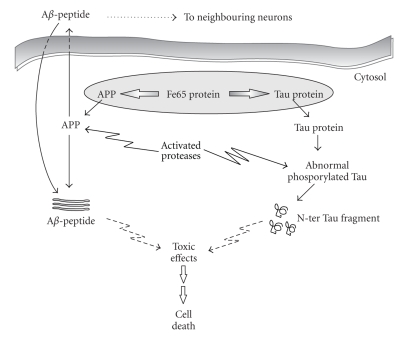
Schematic overview of A*β* peptide and Tau fragments production in CGNs. A*β* is produced intracellularly or taken up from extracellular sources and together with Tau fragments has various pathological effects on cell function.

Interestingly, in the experimental model of CGNs, it has been proposed that Tau and APP form a complex *in vivo* via the adaptor protein Fe65 [33] which is abundantly expressed in the central nervous system of mammals and in particular in the cerebellum and hippocampus [34]. As a consequence, the full-length Tau can play a role in regulating the proper localization of APP and of its partners. During apoptosis, the disruption of the Tau-Fe65 interaction leads to a mislocalization of the APP-Fe65 complex within the cell that in turn could induce a change in the proteolytic fate of both APP and Tau proteins (Figure 1). 

As far as A*β* production is concerned, it has been reported that in the commitment phase (6 hours) of CGN apoptosis, an amyloidogenic process is activated which rapidly and irreversibly leads to increased production of A*β* [27]. A*β* may be released outside the cell and act as a soluble and diffusible apoptotic death mediator, affecting neighbouring healthy neurons and activating a toxic loop that further accelerates and propagates the process of neurodegeneration. Accordingly, it has been found that coincubation of CGN apoptotic cultures with antibodies directed against A*β* significantly slows down the extension of cell death and quantitatively increases the neuronal survival rate [27]. Studies carried out on CGNs as well as on various cell models indicate that both nonaggregated and, to a greater extent, aggregated A*β* peptides of the short toxic fragment A*β*25–35 can induce apoptosis when externally added to cell cultures [35, 36] and that different A*β* aggregation forms (monomers, protofibrillar intermediate, and mature fibrils) can have diverse effects [37–39]. 

In the same experimental model (i.e., CGNs), A*β*25–35-induced apoptosis has been found to be associated with the activation of multiple executioner caspases (caspases-2, -3, and -6) [40], and the shorter A*β* fragment (A*β*31–35) is able to induce neurodegeneration with an early increase in bax mRNA level followed by delayed caspase-3 activation [41]. Finally, it has been reported that A*β* may interfere with K^+^ channel trafficking [42, 43], altering K^+^ currents and therefore causing an increase in cell death as a result of a decrease in cytoplasmic K^+^ concentrations. Consistently, the selective upregulation of the expression of two voltage-dependent potassium channel subunits (Kv4.2 and Kv4.3) has been found in CGNs after A*β*25–35 exposure [44].

In CGNs, contextually to the significant increase in amyloidogenic metabolism of APP, Tau also undergoes posttranslational modifications. As soon as 6 hours after apoptosis induction, a change in Tau phosphorylation state occurs in concomitance with caspase and calpain-mediated cleavages (Figure 1). As a consequence, several fragments of Tau protein are produced during apoptosis, the most abundant of which is a 17 kDa residual fragment, probably located at the NH_2_-terminus of Tau, which is unable to bind to microtubules and is diagnostic for the ongoing apoptotic process [28]. 

Truncated forms of Tau, besides being produced during apoptosis, can also be effectors of apoptosis by themselves and operate as toxic fragments that further induce cell death so contributing to the progression of neurodegeneration by an “autocatalytic process” [29, 45–47]. Both C-ter and N-ter Tau fragments have been analyzed for their neurotoxicity. While the microtubule-binding capacity of the C-ter fragment is well documented, relatively little is known about the function of the N-terminal domain. Transfection of neuronal cells with C-terminal Tau fragments induces cell death [46, 47] while exogenous overexpression of N-ter Tau fragments in CGNs can be either neuroprotective or neurotoxic depending on its length [29]. The long N-ter Tau fragment (1–230) is antiapoptotic and promotes the prosurvival effect of the AKT pathway. On the other hand, the short N-ter Tau fragment (1–44) exerts a toxic action involving glutamate receptors. Moreover, further analysis performed in the CGN model system further narrowed the extent of the aminoacid stretch which is toxic to the cells, and the N-ter-26–44 Tau fragment was found to be the minimal active moiety which retained a marked neurotoxic effect. On the other hand, the NH_2_-1–25 Tau fragment was inactive [48].

## 4. *A* *β* and N-ter Tau Fragments Interaction with Mitochondria

Mounting evidence indicates that mitochondrial dysfunction occurs early in AD, worsens with clinical deterioration, and is associated with impairment of energy homeostasis; deficit in the function of complexes of the respiratory chains reduced ATP synthesis as well as altered mitochondrial structure [49–51]. Consistently, a reduced activity of the cytochrome *c* oxidase (Complex IV of respiratory chain) has been reported in different brain regions [51] as well as in platelets [52] and fibroblasts [53] of AD patients, but the involvement of other mitochondrial oxidative phosphorylation complexes is less documented and more controversial. Cardoso and collaborators [54] found a decreased ATP level in AD cybrids, and other authors report that the activity of Complex IV, but not the activity of F1F0-ATPase (Complex V), decreases in the hippocampus and platelets of AD cases [55, 56]. Because mitochondria are the powerhouse of cells, damage to mitochondria, such as impairment of Complex IV activity, could have functional consequences on energy metabolism [56].

**Figure 2:**
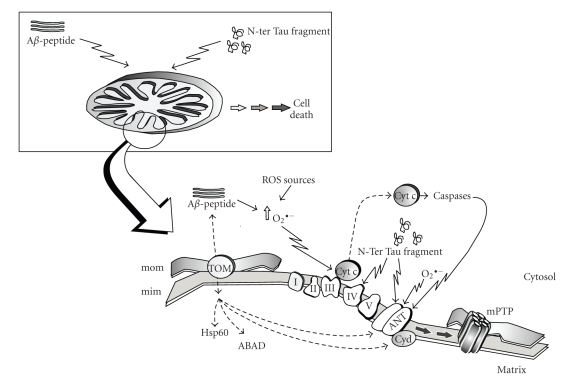
Proposed mechanism of A*β* peptide and N-ter Tau fragment interaction with mitochondria; for further details see text. *mom*, mitochondrial outer membrane; *mim*, mitochondrial inner membrane; *TOM*, translocase of the outer membrane; *I–V*, respiratory chain complexes; *cyt c*, cytochrome *c*; *ANT*, adenine nucleotide translocator; *CyD*, cyclophilin D; *mPTP*, mitochondrial permeability transition pore.

Furthermore, mitochondrial dysfunction has been proposed to be the link between the histopathological hallmarks of AD, caused by A*β* and Tau deposition, and neuronal and synaptic loss [57]. The emerging picture is one in which, at the level of mitochondria, both Alzheimer's proteins exhibit synergistic effects finally leading to the acceleration of neurodegenerative mechanisms (Figure 2).

As far as A*β* is concerned, although the classical view is that A*β* is deposited extracellularly, both cellular and biochemical studies carried out in different models of AD and aging have provided evidence that this peptide can also accumulate inside neurons, target mitochondria, and contribute to disease progression [58–61]. By using in vivo and in vitro approaches, it has been demonstrated that A*β* is transported into rat mitochondria via the translocase of the outer membrane (TOM) [62] and localizes within the mitochondrial cristae. A similar distribution pattern of A*β* in mitochondria has been shown by immunoelectron microscopy in human cortical brain biopsies [62]. 

Interaction of A*β* with mitochondria could be considered a general route common to different cell types since both in dividing cells (i.e., neuroblastoma cells) and in terminally differentiated neurons (i.e., primary neuronal cultures), either extracellulary applied or secreted *A* *β* can be internalized, and it colocalizes with mitochondrial markers [62, 63] (Figure 2). Interaction of A*β* with the matrix protein ABAD (amyloid-binding alcohol dehydrogenase) has been described [64], whereas Caspersen et al. [65] reported that in mouse and human brain samples from AD patients, A*β* colocalizes with the mitochondrial matrix protein Hsp60. Recent biochemical studies imply that the formation of the mitochondrial permeability transition pore (mPTP) is involved in A*β*-mediated mitochondrial dysfunction [66], and by using a computational approach and predictive analysis tools, it has been hypothesized that A*β* can strongly interact in the inner membrane with ANT and Cyclophilin D, two components of the mPTP [67]. 

A connection between Tau protein and mitochondria has recently been proposed; by overexpressing the N-ter Tau fragment truncated at Asp-421 to mimic caspase cleavage in immortalized neurons, it was possible to induce mitochondrial fragmentation and elevated oxidative stress levels [68]. 

To the best of our knowledge, the toxicity of N-ter Tau fragments on mitochondria has been deeply investigated only in the CGN model system and has been found to involve a mitochondrial dysfunction with impairment of oxidative phosphorylation [30] (Figure 2). Both Complex IV and ANT proved to be targets of the short NH_2_-26–44 Tau fragment, but ANT is the only mitochondrial target responsible for the impairment of oxidative phosphorylation. Detailed biochemical studies have revealed that inhibition of ANT is noncompetitive, suggesting that the NH_2_-26–44 Tau fragment does not interact with the catalytic site but with some other site of the enzyme which could distort the enzyme structure thus also affecting the catalytic binding site. 

This finding is consistent with the picture of the apoptotic process in CGN that to date has been built up: in late apoptosis, a noncompetitive-like inhibition of ANT has been found, probably due to caspase activity [26], but it is not dependent on a direct caspase–ANT interaction. However since NH_2_-26–44 Tau fragment is likely to be generated during apoptosis given that the N-terminal domain of Tau contains consensus sequences suitable for cleavage by caspase(s) [28, 45], which are activated in apoptotic degenerating neurons in AD [69, 70], the possibility exists that caspase(s) gradually inhibit/s ANT as a result of NH_2_-Tau cleavage and the generation of toxic NH_2_-26–44 Tau fragment. In this case, NH_2_-26–44 Tau fragment should directly bind ANT.

## 5. Nitric Oxide and AD: Interplay between Alzheimer's Proteins, Nitrosative/Oxidative Stress, and Mitochondria

NO produced by NOS, is a molecule endowed with a double role acting as either a prosurvival or a toxic molecule. As a prosurvival molecule, NO plays a role in cell signaling in the nervous system and in synaptic plasticity [71, 72], and it may be involved in diverse biological functions acting through either cGMP-dependent or -independent pathways. 

**Figure 3:**
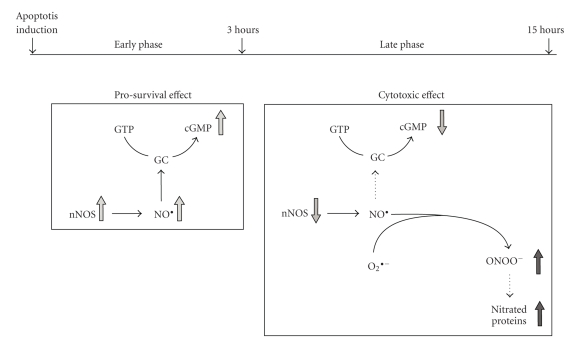
Schematic representation of the time-dependent, dual role of nitric oxide in CGN apoptosis; see text for details. *GC*, guanylil cyclase; *cGMP*, cyclic GMP; *nNOS*, neuronal nitric oxide synthase; *O*_2_^∙^^−^, superoxide anion; *O* *N* *O* *O* ^−^, peroxynitrite.

When the role of the NO/NOS system was investigated in CGNs, it was found that NO exerts its dual and opposite effects on the neurodegenerative process, depending on the time after induction of apoptosis (Figure 3). In an early phase, up to 3 h of apoptosis, there is an increase in the expression of the neuronal isoform of NOS (nNOS) as well as in the production of NO, which in turn supports the survival of CGNs through a cGMP-dependent mechanism. 

Consistently with these results, it has also been reported that: (i) NO may be responsible for neuroprotection during A*β*-induced cell death [73, 74], (ii) low concentration of NO produced by a healthy cerebrovascular endothelium was found to influence the parenchymal brain cells in a protective way [75], and (iii) in cultured human neuroblastoma cells, low concentrations of NO upregulate the expression of alpha-secretase, while downregulating that of beta-secretase, suggesting that, in the relative absence of superoxide, cerebrovascular NO might act to suppress brain production of A*β* [76]. 

On the other hand, sustained generation of NO has been implicated in the cellular death occurring in different neurodegenerative diseases as well as in AD [77]. As far as the experimental system of CGNs is concerned (Figure 3), it was found that, in the late phase of the apoptotic program, after 3 h, nNOS expression and activity decreased, resulting in the shut down of NO and cGMP production, and the toxic role of nitric oxide prevailed due to the reaction with superoxide anions to produce peroxynitrite (ONOO^−^) which in turn is able to induce neuronal injury mainly through nitration of tyrosine residues in cellular proteins, whose level increases. These events together with other apoptotic events already described in this cell model [15, 23, 25, 26] would commit these cells irreversibly to death. 

**Figure 4:**
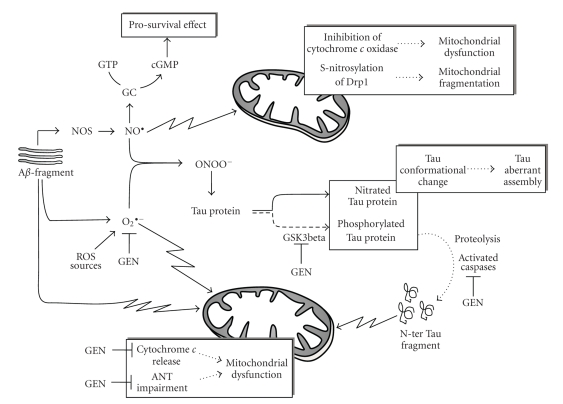
Schematic overview of the interplay between A*β*, Tau, oxidative/nitrosative stress, and mitochondria; see text for details. *GC*, guanylil cyclase;* cGMP*, cyclic GMP; *NOS*, nitric oxide synthase; *O*_2_^∙^^−^, superoxide anion; *O* *N* *O* *O* ^−^, peroxynitrite; *ANT*, adenine nucleotide translocator; *GSK3beta,* glycogen synthase kinase-3beta; *GEN*, Genistein.

Thus, it can be assumed that once accumulated inside the cell, NO can play different roles, depending on its level, cell context, and amount of superoxide anion. In Figure 4, a general picture is shown which takes into account the main findings on the involvement of nitrosative stress in the neurodegenerative process. In brains from AD patients, an early and striking upregulation of all three isoforms of NOS has been reported [78, 79]. This finding is further supported by experimental data obtained in different systems, ranging from in vivo animals to cell lines, which indicates that NO is responsible for A*β* toxicity and highlights a link between NO/NOS level and A*β*-induced brain dysfunction [80, 81]. Activation of the neuronal isoform of NOS (nNOS) [82] and an increased production of NO [83] were also found in rat cerebral cortex and hippocampus after intracerebroventricular administration of A*β*25–30 and in APP-transfected cells, respectively. 

In an early phase, NO could induce a cGMP-mediated prosurvival signaling pathway in an attempt to counteract the ongoing neurodegenerative process [19, 84]. However, NO can also directly trigger mitochondrial dysfunction, a process which is believed to play a causative role in AD onset and progression. Indeed it has been reported that NO both induces a bioenergetic failure, with impairment in the function of Complex IV [85], and triggers mitochondrial fission/fragmentation thus causing cell death in primary culture of cortical neurons [86, 87]. S-nitrosylation, a covalent redox reaction of NO with specific protein thiol groups, could be one mechanism contributing to the NO-induced mitochondrial fragmentation. Accordingly, it has been reported that in AD patients and animal model, NO induces S-nitrosylation of dynamin-related protein 1 (Drp1), a protein specifically involved in mitochondrial fission [88, 89]. On the other hand, Bossy et al. [90] found that NO can also induce Drp1 inactivation by increasing its phosphorylation. Although there are no data on the involvement of Drp1 in the CGN model, it has been recently reported that mitochondrial fragmentation occurs as an early event in response to injury in CGNs, and increased activation of mitofusin 2 (Mfn2), a protein involved in mitochondrial fusion, blocks mitochondrial fragmentation and protects neurons against cell death [91, 92].

In addition to NO, oxidative damage has been reported in aging and age-related neurodegenerative diseases, including AD [93, 94], and superoxide anion production has been induced by A*β*-treatment in neurons [95, 96]. It is known that in the course of neurodegeneration, the superoxide anion can act directly on mitochondria thus inducing cyt *c* release and precocious impairment of ANT (see [18] and references therein).

On the other hand, NO readily reacts with superoxide anions to form the strong oxidant ONOO^−^ which in turn induces protein nitration. Consistently, an increase in protein nitration has been found in brain tissue from cases of AD which correlates with neurodegeneration [97]. Tau protein can also undergo a ONOO^−^-mediated process, and nitration of the Tyr29 residue has been proposed as a specific disease-related event [98]. Furthermore, peroxynitrite can also induce AD-like Tau hyperphosphorylation via activation of both glycogen synthase kinase-3beta (GSK3beta) and p38 MAPKs [99]. 

Nitration, as well as phosphorylation, of Tau protein induces conformational changes that facilitate aberrant Tau assembly. Consistently, it has been reported that nitrated Tau is colocalized with neurofibrillary tangle in AD brain, shows a significantly decreased binding activity to microtubules, and is involved in the formation of filamentous Tau inclusions [100]. In these conditions, Tau fragmentation might occur, and N-ter Tau fragments, together with A*β* and superoxide, can further decrease mitochondrial efficiency thus contributing to mitochondrial dysfunction.

## 6. Implication of Genistein on Preventing *A* *β* and Tau Toxicity

The main goal in AD treatment is focused on a preventive approach. Treatment of declared AD with any compounds may have either a poor effect due to the severe neuronal death occurring in AD or a questionable risk/benefit ratio such as in the case of estrogen. In this regard, estrogen has been shown to block A*β*-induced neuronal cell death in several studies thus suggesting that estradiol replacement therapy should show improvement in patients with AD [101]. However, the efficiency of estradiol in the treatment of AD has been seriously questioned due to its fourth unwanted side effect, that is, proliferative and oncogenic effects on non-neuronal cells [102]. 

A clear point emerging from the bulk of studies dealing with AD etiopathology is that all factors involved in AD are associated with oxidative stress [103]. In the light of this, natural oxidants have recently received much attention as promising agents for reducing the risk of oxidative stress-related diseases. Among them genistein received a lot of attention. 

Genistein (4′.5.7-trihydroxyisoflavone) is the most active compound of soy isoflavones, the one which reaches the highest concentration in human blood [104], possesses an antioxidant activity, shows an affinity to estrogen receptors, thus acting as an estrogen-like compound but without the negative effects of estrogens, and is able to cross the blood-brain barrier (see [105]).

There is considerable literature about the effect of genistein on the progression of neurodegeneration. It has been reported that in the nervous system, isoflavones attenuate primary neuronal apoptosis by activating estrogen receptors [106] and genistein is able both to suppress A*β*25–35-induced ROS overproduction in isolated rat brain synaptosomes [107] and to increase cell viability in cooperation with other trophic factor such as folic acid in cortical neurons [108]. Consistently, Zeng et al. [105] describe the protective effect of genistein on cultured hippocampal neurons against A*β*-induced apoptosis and have demonstrated that genistein inhibits the elevation of intracellular free Ca^2+^, the production of oxidant free radicals caused by A*β*25–35, the DNA fragmentation, and the activation of caspase-3, thus suggesting that genistein acts upstream of caspase-3 to block apoptosis (Figure 4).

Genistein may also decrease the hyperphosphorylation of Tau protein by inactivating GSK3beta, the kinase involved in Tau phosphorylation in homocysteine-mediated neurodegeneration in SH-SY5Y human neuroblastoma cells [109].

Recently, in CGNs undergoing apoptosis, the effect of genistein was studied at subcellular level and for the first time at mitochondrial level [110]. Genistein and to a lesser extent its analogue daidzein, both used at dietary concentrations, can prevent low potassium-dependent apoptosis in CGNs by reducing the impairment of both aerobic glucose metabolism and mitochondrial uncoupling, two processes occurring in CGN apoptosis [16]. Furthermore, genistein is also able to prevent cyt *c* release, ANT alteration, and mPTP opening; that is, some steps of the mitochondrial pathway to apoptosis that are somehow related to the ROS production which takes place during apoptosis. 

Thus, since both genistein and daidzein have been proved to decrease ROS levels, it has been suggested that the prevention of apoptosis is essentially due to the antioxidant properties of these flavonoids [110]. Nonetheless, the effect of genistein proved to be rather specific since other flavonoids such as catechin and epicatechin failed to prevent CGN death in spite of their shared antioxidant capability. 

Consistently, genistein also abolishes neuronal ROS production induced by *A* *β* administration to primary culture of cortical neurons [111] and enhances the activities of other antioxidant molecules and enzymes (superoxide dismutase, glutathione peroxidase and reductase) both in vitro and in vivo [112, 113].

## 7. Conclusions

The etiology of Alzheimer's disease is complex and not fully elucidated. On the other hand, it is important to develop a better understanding of the different biochemical pathways, their role, and their link with the amyloid hypothesis in AD, since it may lead to the development of more effective treatment strategies for this disease. It seems clear then that promising developments as for the prevention and/or delay of the onset of AD can be derived from definition of antiapoptotic treatments acting on the precocious steps of the death process, such as blockade of generation of reactive oxygen species and implementation of the NO prosurvival signaling pathway that, although not able to fully prevent the disease, can at least delay onset or reduce the severity of neurodegeneration. In this regard, genistein and its analogue daidzein may perhaps be of use in neuroprotection. Furthermore, the knowledge emerging from studies conducted on CGNs, that ANT impairment contributes in a significant manner to bioenergetic failure and mitochondrial dysfunction in the course of neurodegeneration, may open a window for new therapeutic strategies aimed at preserving and/or improving mitochondrial function, representing an exciting challenge for biochemists. More studies are required to determine whether phytoestrogens, protease inhibitors and mitochondrial-targeted compounds could fulfill these expectations.
